# Gene editing in CHO cells to prevent proteolysis and enhance glycosylation: Production of HIV envelope proteins as vaccine immunogens

**DOI:** 10.1371/journal.pone.0233866

**Published:** 2020-05-29

**Authors:** Sophia W. Li, Meredith Wright, John F. Healey, Jennie M. Hutchinson, Sara O’Rourke, Kathryn A. Mesa, Pete Lollar, Phillip W. Berman

**Affiliations:** 1 Department of Chemistry and Biochemistry, University of California Santa Cruz, Santa Cruz, California, United States of America; 2 Department of Biomolecular Engineering, University of California Santa Cruz, Santa Cruz, California, United States of America; 3 Department of Pediatrics, Emory University, Atlanta, Georgia, United States of America; Translational Health Science & Technology Institute, INDIA

## Abstract

Several candidate HIV subunit vaccines based on recombinant envelope (Env) glycoproteins have been advanced into human clinical trials. To facilitate biopharmaceutical production, it is necessary to produce these in CHO (Chinese Hamster Ovary) cells, the cellular substrate used for the manufacturing of most recombinant protein therapeutics. However, previous studies have shown that when recombinant Env proteins from clade B viruses, the major subtype represented in North America, Europe, and other parts of the world, are expressed in CHO cells, they are proteolyzed and lack important glycan-dependent epitopes present on virions. Previously, we identified C1s, a serine protease in the complement pathway, as the endogenous CHO protease responsible for the cleavage of clade B laboratory isolates of -recombinant gp120s (rgp120s) expressed in stable CHO-S cell lines. In this paper, we describe the development of two novel CHOK1 cell lines with the C1s gene inactivated by gene editing, that are suitable for the production of any protein susceptible to C1s proteolysis. One cell line, C1s^-/-^ CHOK1 2.E7, contains a deletion in the C1s gene. The other cell line, C1s^-/-^ MGAT1^-^ CHOK1 1.A1, contains a deletion in both the C1s gene and the MGAT1 gene, which limits glycosylation to mannose-5 or earlier intermediates in the N-linked glycosylation pathway. In addition, we compare the substrate specificity of C1s with thrombin on the cleavage of both rgp120 and human Factor VIII, two recombinant proteins known to undergo unintended proteolysis (clipping) when expressed in CHO cells. Finally, we demonstrate the utility and practicality of the C1s^-/-^ MGAT1^-^ CHOK1 1.A1 cell line for the expression of clinical isolates of clade B Envs from rare individuals that possess broadly neutralizing antibodies and are able to control virus replication without anti-retroviral drugs (elite neutralizer/controller phenotypes). The Envs represent unique HIV vaccine immunogens suitable for further immunogenicity and efficacy studies.

## Introduction

The majority of recombinant glycoprotein therapeutics are manufactured in CHO (Chinese Hamster Ovary) cells due to their high productivity (1–10 grams per liter), genetic stability, and ability to be grown in large-scale suspension culture [1–3]. However, many recombinant proteins including monoclonal antibodies, antibody fusion proteins, and IFN-γ are partially degraded or “clipped” by endogenous CHO cell proteases during the cell culture or recovery process [4–9]. This is also the case for glycoprotein 120 (gp120), the monomeric subunit of the HIV-1 envelope protein (Env), used in many of the HIV vaccines tested to date in human vaccine efficacy trials [10–13]. The HIV Env protein mediates virion binding to CD4, the T-cell surface receptor, and to the CXCR4 or CCR5 chemokine receptors [14–16]. Env proteins have been included in most HIV vaccines since they are the major target for virus neutralizing antibodies [17–19]. HIV isolates are classified into different genetic clades based on impartial sequence analysis [20,21]. These include clades C and CFRF01_AE viruses, prevalent in Africa and Asia respectively, and clade B viruses in North America, Europe, the Caribbean and Australia. Because they lack the clade B consensus sequence Gly-Pro-Gly-Arg-Ala-Phe (GPGR/AF) at the crown of the V3 domain, most clade C and CRF01_AE Envs can be produced in CHO cells without proteolysis. In contrast, the V3 domain of most clade B Envs has been shown to be highly sensitive to proteolysis by exogenous thrombin or an unidentified CHO cell protease [22–25]. Env proteins proteolyzed in this manner are difficult to manufacture and purify in quantities required for immunization of populations at high risk for infection [24,26].

Recently, we reported that the major CHO cell protease responsible for cleavage of clade B gp120s was the complement component 1 protease, C1s [27]. C1s is a serine protease that recognizes the sequence Gly-Pro-Gly-Arg, located in the V3 loop of gp120. This sequence is present in 71% of clade B HIV strains [28] and is also present in the Env protein from the clade A/G Z321 isolate, one of the earliest known strains of HIV [29]. The V3 loop mediates binding to the coreceptors, CXCR4 or CCR5 [30]. Thus, antibodies to this portion of the V3 region are highly effective at virus neutralization. These antibodies include the monoclonal antibody (mAb), 447-52D, that binds to the crown of the V3 region [31], and the glycan-dependent, broadly neutralizing monoclonal antibodies (bN-mAbs), PGT121, PGT128, and 10–1074, that bind to the stem of the V3 region [32–35]. Because the GPGR/AF sequence is part of, or adjacent to, the epitopes recognized by these neutralizing antibodies, it is necessary to keep the V3 loop intact on the HIV Env immunogen in the hopes of eliciting similar, neutralizing antibodies. In our previous study, we showed that CRISPR/Cas9 inactivation of the C1s gene in a stable CHO-S cell line expressing gp120 from the laboratory-adapted isolate, HIV_BaL_, prevented proteolysis at the GPGPR/AF sequence in the V3 domain. As this cell line is uniquely developed to express BaL-rgp120, it cannot be used for the expression of other recombinant proteins. Therefore, a C1s knockout CHO cell line, that can be used as the cell substrate for the expression of any other recombinant protein, is needed.

In this paper, we describe the development of two novel, suspension-adapted CHOK1 cell lines useful for the production of biopharmaceutical proteins. The first cell line, C1s^-/-^ CHOK1 2.E7 is a CHOK1 cell line where the C1s gene has been inactivated by gene editing. The second cell line, C1s^-/-^ MGAT1^-^ CHOK1 1.A1 is also a CHO K1 cell line where both the C1s and MGAT1 genes have been inactivated. Inactivation of the MGAT1 gene limits N-linked glycosylation to mannose-5 and earlier intermediates in the N-linked glycosylation pathway. Limiting N-linked glycosylation via the MGAT1 cell line has been shown to improve the antigenic structure of gp120 as measured by the ability to bind multiple bN-mAbs [36–38].

To demonstrate the utility of these cell lines, we expressed three novel clinical isolates of clade B gp120s that have potential as improved HIV vaccine immunogens. These gp120s were derived from rare individuals, who either possessed broadly neutralizing antibodies (elite neutralizers) and/or were able to control their virus replication for extended periods of time without anti-retroviral drug treatment (viral controllers and elite controllers) This will allow us to further test the hypothesis that Env proteins from individuals with the elite neutralizer and/or rare controller phenotype possess unique structural features that are particularly effective in stimulating effective, anti-viral immune responses. Additionally, we studied the expression of another recombinant protein, human Factor VIII, known to be susceptible to proteolysis in CHO cells to explore the substrate specificity of C1s and how it differs from thrombin, another enzyme able to cleave the GPGRAF consensus sequence. This work documents the C1s^-/-^ CHOK1 cell line and C1s^-/-^ MGAT1^-^ CHOK1 cell line as novel cell substrates for the production of biopharmaceutical recombinant proteins.

## Materials and methods

### Antibodies and purified proteins

The coding sequences of the PG9, PGT128, VRC01 bN-mAbs were obtained from published sequences (available from the NIH AIDS Reagent Program, Germantown, MD) and used to created synthetic genes for stable or transient expression of antibodies in CHO cells. Antibodies were purified using Protein G chromatography. These cell lines have been deposited in the NIH AIDS Reagent Program. The 10–1074 bN-mAb was acquired from the NIH AIDS Reagent Program. The 34.1 mAb to the Herpes Simplex Virus glycoprotein D (gD) was produced in-house [39]. The human 447-52D mAb was contributed by Dr. Susan Zolla-Pazner (Mount Sinai, New York City, NY). Recombinant rgp120s were cloned with an N-terminal gD tag into a pCF vector [39]. Plasmids were transformed in 5-alpha competent E. coli cells (New England Biolabs, Ipswich, MA), purified using Plasmid Maxi Kit (Qiagen, Hilden, Germany) and electroporated into CHO cells as described below. Recombinant human factor VIII consisted of Advate (Baxter Healthcare Corporation, Deerfield, Illinois) [40], and was acquired by the laboratory of Dr. Peter Lollar (Emory University, Atlanta, GA).

### Clade B clinical isolates

Genes encoding gp120s were cloned from cryopreserved plasma and/or peripheral blood mononuclear cells from anti-retroviral therapy naïve individuals. Clade B Envs (EN2, EN3, EN6, and EN7) were obtained from individuals prescreened for the presence of bN-mAbs and the ability to control virus loads and CD4 counts without antiretroviral therapy. The clade B isolates were obtained from the SCOPE Cohort (Observational Study of the Consequences of the Protease Inhibitor Era cohort) and WIHS cohort (Women’s Interagency Health Studies cohort). The Envs were screened for the presence of bN-mAbs using the Simek panel of HIV pseudoviruses [41] and individuals with high levels of bN-mAbs were selected for further study. In these studies, Envs were amplified by PCR and screened for infectivity by Monogram Biosciences (South San Francisco, CA) using the Phenosense assay system.

### Culture of CHOK1 cells

CHO K1 cells (ATCC^®^ CCL-61) were obtained from the American Type Culture Collection (ATCC, Manassas, VA) and were adapted to suspension culture as described previously [37]. These were maintained in shake flasks (Corning, Corning, NY) using a Kuhner ISF1-x shaker incubator (Kuhner, Birsfelden, Switzerland) at 37°C, 8% CO2, and 125 rpm. Static cultures were maintained in 96 or 24 well cell culture dishes (Corning) and grown in a Sanyo incubator (Sanyo, Osaka, Japan) at 37°C and 8% CO2. All cell counts were performed using a TC20TM automated cell counter (BioRad, Hercules, CA) with viability determined by trypan blue exclusion (Thermo Fisher Scientific).

For transient protein expression, plasmid DNA was eluted into endotoxin-free water at concentrations greater than 5 mg/ml for optimal transfection efficiency by electroporation using the OC-400 cuvette and the MaxCyte transfection system (MaxCyte, Gaithersburg, MD) as previously described [37]. 1M Sodium Butyrate was added to a final concentration of 1 mM to limit cell doubling. Cells underwent a temperature shift from 37C to 34C. Cells were maintained in CHO Growth A media and 8mM glutamine (GlutaMAX, Gibco) and additionally supplemented at 10% of culture volume every 3 days with a feed consisting of Proyield Cotton CNE50M-UF protein hydrolysate (FrieslandCampina, Amersfoort, Netherlands), CD Efficient Feed C (Gibco) and 16mM glutamine.

For normal cell growth, cells were maintained in serum free CHO Growth A media (Irvine Scientific, Santa Ana, CA) supplemented with 8mM GlutaMAX (Gibco, Gaithersburg, MD). Cells were initially maintained in serum free CD CHO Medium (Gibco) with 8mM GlutaMAX. However, due to clumping of cells, the media was switched to CHO Growth A. The doubling time for cells was calculated using the formula for population doubling time, DT = (T ln 2)/(ln (Xe/Xb)) where T is the incubation time, Xe is the number of cells at the end of the incubation time and Xb is the number of cells at the beginning of the incubation time.

### Detection of secreted proteins by immunoblot

The apparent molecular mass of either purified gp120 or unpurified gp120s in cell culture supernatants was determined by sodium dodecylsulfate polyacryamide gel electrophoresis (SDS-PAGE) on NuPAGE 4–12% Bis-Tris precast gels (Thermo Fisher Scientific, Invitrogen, Carlsbad, CA). Samples reduced by dithiothreitol (DTT) were run on gels in MES (2-(N-morpholino)ethanesulfonic acid) running buffer (Thermo Fisher) and stained with SimplyBlue stain (Thermo Fisher) For immunoblots, PAGE gels were transferred using iBlot 2 (Thermo Fisher). Membranes were blocked with 5% milk for 1 hour. The primary antibody, a polyclonal goat antibody raised against gp120, was incubated with the membrane at 1.5 ug/ml concentration in 5% milk. Blots were washed three times with PBS with 0.05% Tween (PBS-T). The secondary antibody used was the Peroxidase AffiniPure bovine anti-goat IgG (Jackson ImmunoResearch, West Grove, PA) and incubated with the membrane at a 1:5000 dilution in 5% milk. Blots were washed three times with PBS-T and three times with PBS. The chemiluminescent substrate used was WesternBright ECL horseradish peroxidase substrate (Advansta, Menlo Park, CA). Images were taken using a FluorChem Q imager (Alpha Innotech, San Leandro, CA).

### Fluorescence immunoassay (FIA)

A fluorescent immunoassay (FIA) was used to measure gp120 concentrations. In this assay, Fluortrac high binding 96 well plates (Grenier Bio One, Kremsmunster, Austria) were coated overnight at 4°C with 2 ug/ml of 34.1 mAb. Plates were blocked for 5 hours with 2% BSA and washed 4x with PBS-T. Growth-conditioned cell culture supernatant was diluted in 1% BSA/PBS-T and incubated overnight at 4°C. Antibodies were 3x serially diluted from 10 ug/ml to 0.003 ug/ml and incubated for 1.5 hours. After a 4x wash with PBS-T, secondary antibodies were added at a 1:3000 dilution in 1% BSA/PBS-T and incubated for 1.5 hours. Alexa Fluor 488 Goat α-human IgG (Jackson ImmunoResearch), Donkey α-goat IgG (Invitrogen), or Goat α-mouse (Invitrogen) IgG were used. After a 4x PBS-T wash, wells were filled with 50 ul PBS. Plates were read on an EnVision plate reader (Perkin Elmer Inc, Waltham, MA) at excitation/emission wavelengths of 353/485 nm. All assays were performed in triplicate.

### CRISPR/Cas9 knockout of the C1s^-/-^ gene in the CHOK1 cell line and the MGAT1^-^ CHOK1 cell line

The C1s gene was isolated from CHO cells by PCR as described previously [27] sequenced, and verified against the NCBI mRNA Reference Sequence XM_007646821.2 in Cricetulus griseus and the predicted protein NCBI Reference Sequence XP_007645011.1. For gene inactivation, the guide RNA, GTTGACAGCCGCTCATGTTG, was synthesized and cloned into the CRISPR Nuclease Vector (GeneArt, Thermo Fisher) which expresses Cas9 and an orange fluorescent protein reporter. To ligate sgRNA inserts into the vector, DNA oligos were synthesized (Eurofins Genomics, Louisville, KY). Bacterial amplification of the completed vectors was carried out in One Shot TOP10 Chemically Competent E. coli following the recommended protocol (Thermo Fisher). Culture were grown in 15 ml LB broth overnight at 37°C at 250 rpm. Minipreps (Qiagen, Redwood City, CA) were performed using the recommended protocol and submitted for Sanger sequencing (University of California Core Sequencing Facility, Berkeley, CA) with the U6 primer provided in the GeneArt CRISPR kit. 1 liter Maxipreps (Qiagen) were performed using the recommended protocol. Plasmid DNA was eluted into endotoxin-free water at concentrations greater than 5 mg/ml for optimal transfection efficiency by electroporation using the MaxCyte transfection system as previously described [37].

For single cell cloning, five 96 well flat-bottomed tissue-culture treated microplates were filled with 50 ul of conditioned serum free CHO Growth A media. Cells were serially diluted to 10 cells/ml in fresh CHO Growth A media and 50 ul of the diluted cells were added to each well, for a final concentration of 0.5 cell/well. Growth was monitored daily; any wells with more than a single colony were discarded. Cells were grown for 20 days at 37C, 8% CO2 and 85% humidity. At 50% confluency, cell culture supernatants from each clone were mixed with exogenous gp120 and analyzed by immunoblot to observe proteolytic activity. For the immunoblot, 200 ng of purified, BaL-gp120 was incubated with 16 ul of supernatant at 37°°C for 48 hours. Cell culture supernatants from clones that were unable to cleave purified BaL-rgp120 were then sequenced to verify presence of CRISPR/Cas9-induced indels). To sequence the C1s genes, 0.5 x 10e6 cells were spun down and boiled in 10 ul of milliQ water. 5 ul of cell lysate was used for PCR using OneTaq Hot Start DNA Polymerase (New England Biolabs). Positive clones with confirmed indels in both copies of the C1s gene were moved into 24 well plates in a volume of 150 ul. At 50% confluence, clones were moved into 6 well plate in a volume of 3 ml in a shake flask.

### Proteolysis of recombinant factor VIII and Dylight 650-labeled factor VIII by SDS-PAGE

Recombinant human factor VIII from therapeutic drug, Advate was treated with growth conditioned cell culture supernatant from normal (C1s containing) CHOK1 cell supernatants or supernatants from the C1s^-/-^ CHOK1 2.E7 cell line. For these experiments, Advate was diluted in HBS/C/T-80, FVIII dilution buffer (20 mM HEPES/0.15M NaCl/5mM CaCl2/0.01% TWEEN-80, pH 7.4) to 100 ug/ml. Advate was then diluted with either buffer or CHOK1 cell supernatants at a 1:8 ratio. Samples were incubated at 37C for 24 hours. 5 U/ml of bovine thrombin was incubated with 1 ug Advate as the positive control. 0.4 ug Advate was loaded onto both the SDS-PAGE gel and Dylight-labeled Advate gel. SDS-PAGE gels were stained with GelCode Blue stain (Thermo Fisher). Dylight 650-labeled (Thermo Fisher) FVIII was visualized using a 700 nm channel on an Odyssey imaging system (LI-COR Biosciences, Lincoln, NE).

## Results

### Proteolysis of clade B gp120s in CHO cells

For these studies, we selected clade B Envs from three unrelated individuals with the rare elite neutralizer/controller phenotype, EN2, EN6 and EN7 (Mesa, et al. in preparation, Hutchinson, et al. in preparation), similar to other elite neutralizer/controllers previously described [42,43]. One Env from each individual, was selected for further study based on their bN-mAb-binding ability. The physical and chemical characteristics, organized region by region, for the domain lengths, insertions, deletions and glycosylation sites of the three Envs are summarized (Table 1). The gp120 lengths varied from to 474 to 491 amino acids and the number of predicted N-linked glycosylation sites (PNGS) varied from 17 to 24 PNGS. In contrast, the protease resistant control Envs, A244 and EN3, ranged from 499 to 523 amino acids in length and contained from 20 to 25 PNGS (S1 Fig). The three Envs contain the protease-sensitive motifs Gly-Pro-Gly-Arg, or GPGR, at the crown of the V3 loop [22,27] (Table 2). In contrast, the protease resistant A244 isolate and the clade B EN3 possess GPGQ and GPGG at the crown of the V3 domain, respectively.

**Table 1:** Physical characteristic of elite neutralizer/controller rgp120s used in this study.

		EN2_005		EN6_226		EN7_090	
Domain	HXB2	Length (aa)	PNGS	Length (aa)	PNGS	Length (aa)	PNGS
**Signal**	**1–30**	29	0	29	0	29	0
**C1**	**31–130**	102	2	100	0	100	2
**V1**	**131–157**	35	5	25	1	32	5
**V2**	**158–196**	41	2	38	2	39	2
**V1/V2**	**131–196**	76	7	63	3	71	7
**C2**	**197–295**	99	5	99	5	99	6
**V3**	**296–331**	35	1	35	1	35	1
**C3**	**332–384**	52	2	52	2	52	3
**V4**	**385–418**	32	4	31	4	35	4
**C4**	**419–459**	43	1	41	1	41	0
**V5**	**460–469**	10	2	11	1	14	2
**C5**	**470–511**	42	0	42	0	42	0
**Extracellular**	**512–678**	166	5	166	4	166	4
**Transmembrane**	**679–699**	22	0	22	0	22	0
**Cytoplasmic**	**700–856**	157	1	157	1	157	1
**gp120**	**1–511**	491	24	474	17	489	25
**gp41**	**512–856**	345	6	345	5	345	5
**gp160**	**1–856**	836	30	819	22	834	30

**Table 2:** V3 domain crown sequences for EN rgp120s.

Env	Sequence	Clade	Cleaved by CHO C1s
EN2	GPG**R**VWYA	B	+
EN6	GPG**R**PEGI	B	+
EN7	GPG**R**AFYA	B	+
A244	GPG**Q**VFYR	AE	-
EN3	QPGGAIYA	B	-

The length in amino acids (AA) and number of potential N-linked glycosylation sites (PNGS) are listed for each of the HIV Env domains for the expressed Envs: EN2_005, EN6_226 and EN7_090.

V3 domain crown sequences for the transiently expressed Envs are listed, along with the virus clade and summary of susceptibility to proteolysis by CHO C1s.

**Fig 1:**
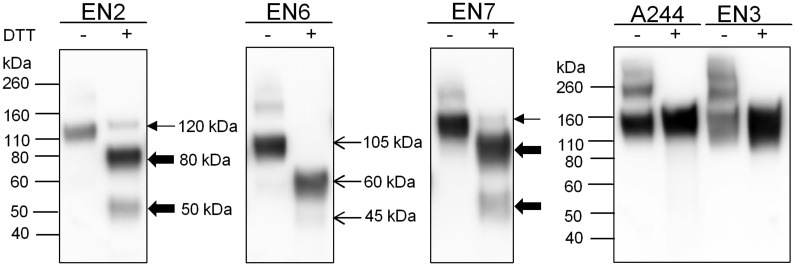
Proteolysis of six HIV Env gp120s expressed in CHO cells as visualized by immunoblot. Clade B HIV Env gp120s from four individuals with neutralization breadth (EN2, EN6, EN7, EN3) were transiently transfected into normal CHOK1 cells. Cell culture supernatant was analyzed by immunoblot, and samples were run non-reduced and reduced with DTT. Full-length gp120 is indicated with thin arrows and the proteolyzed 80kDa and 50 kDa fragments are indicated with thick arrows for EN2 and EN7. For EN6, full-length gp120 runs as 105 kDa due to the presence of only 17 PNGS and has smaller proteolyzed fragments than EN2 and EN7. Blots were probed with a goat polyclonal serum to gp120. The clade CRF01-AE, A244-rgp120 that lacks the clade B GPGRAF consensus sequence was expressed in normal CHOK1 cells and used as a negative control.

When EN2, EN6, and EN7 were transiently transfected in normally CHOK1 cells and analyzed by immunoblot, non-reduced gp120 was observed at 120 kDa (Fig 1). After reduction, additional bands of 80 and 50 kDa bands appeared that were indicative of proteolysis at the V3 domain. The same cleavage pattern occurred with Envs from clade B, BaL and MN and other strains, as was previously observed [24,25,27]. Full-length EN6 has a lower molecular weight of 105 kDa compared to EN2 and EN7 due to significantly fewer glycans and was cleaved into 60 kDa and 45 kDa fragments. Additionally, we examined the protease sensitivity of two Envs (A244 and EN3) that possessed sequences in the V3 crown that differed from the clade B consensus GPGR sequence. The CRF01_AE A244 isolate from Thailand possesses the GPGQ sequence typical of non-clade B and non-clade F2 Envs as shown in the consensus sequences for the clades of Group M (Table 3). Although EN3 is a clade B virus, it possesses an unusual QPGG sequence at the crown of the V3 region. As expected, neither Env was proteolyzed when expressed in CHO cells. This data suggests that there is no difference in accessibility to C1s cleavage in the V3 domain between Envs and that all Envs with the GPGR motif will be subject to proteolysis.

**Table 3:** V3 consensus sequences.

Clade	V3 Consensus Sequence
Con A1	GPG**Q**AFYA
Con A2	GPG**Q**AFYT
Con B	GPG**R**AFYT
Con C	GPG**Q**TFYA
Con D	GPG**Q**ALYT
Con F1	GPG**Q**AFYA
Con F2	GPG**R**AFYA
Con G	GPG**Q**AFYA
Con H	GPG**Q**AFYA
Con AE	GPG**Q**VFYR

HIV Env consensus sequences for the V3 crown are listed for all of the major clades of HIV (from the Los Alamos National Laboratory HIV Sequence Database). Shaded rows have sequences with the GPGR motif recognized by C1s.

### Creation of a CHOK1 cell line deficient in C1s

**Fig 2:**
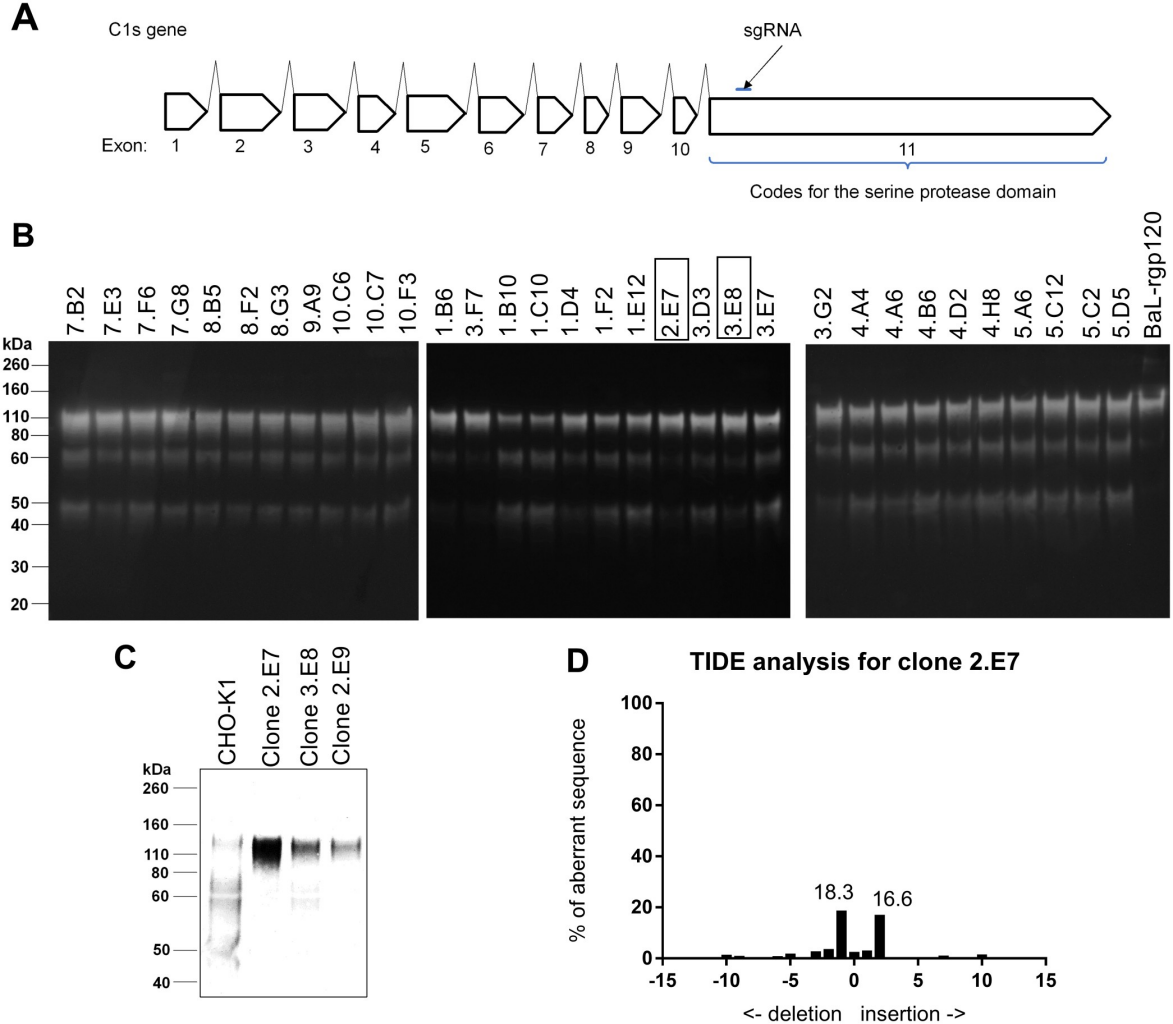
Selection of C1s^-/-^ CHOK1 2.E7. A. A schematic for the C1s gene, containing 11 exons. The single guide RNA targeting exon 11 used to direct Cas9 is indicated by the arrow. B. Clones with both alleles of C1s knocked out were screened by Western Blot. 100 ng of purified BaL-rgp120 was incubated with 15 ul of supernatant from 24 well plates overnight at 37°C. Samples were run reduced with DTT. Clones marked with a black box have knockouts of both alleles as verified by sequencing. C. Bal-rgp120 was transiently transfected into C1s^-/-^ CHOK1 clones 2.E7, 2.E9, and 3.E8 and cultures were grown for 6 days. 5 ul of supernatant was run reduced with DTT on Western Blot. D. The sequence around the expected indel site was analyzed by TIDE (Tracking of Indels by Decomposition, tide.deskgen.com) for clone 2.E7 to check for knockout of both alleles. The indel site was amplified by PCR from genomic DNA extracted from the CHO cell clone and Sanger sequenced. The peaks from Sanger sequencing chromatograms are decomposed to determine the proportion and identity of the indels present in the clone.

**Fig 3:**
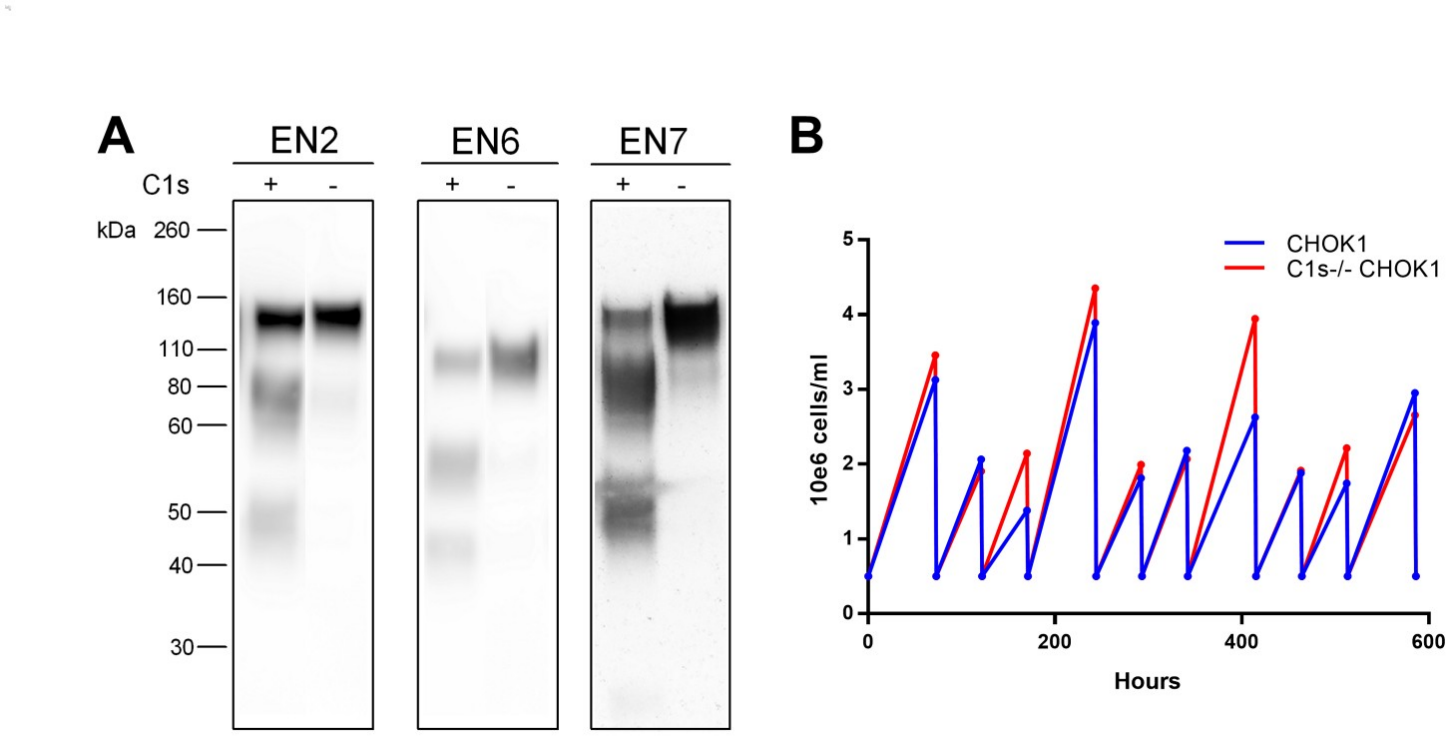
Characteristics of the C1s^-/-^ CHOK1 2.E7 cell line. A. Comparison of proteolysis of clade B gp120s expressed in CHO cells with and without the presence of C1s. EN2, EN6 and EN7 rgp120s were transiently transfected in the CHOK1 and the C1s^-/-^ CHOK1 2.E7 cell line by electroporation. rgp120s expressed in the CHOK1 cell line are indicated with (+) and those expressed in the C1s^-/-^ CHOK1 2.E7 cell line are indicated with (-). Supernatants were analyzed by immunoblot using an anti-rgp120 polyclonal antibody. Cell culture supernatant was assayed by immunoblot with a goat polyclonal serum to gp120. B. The viable cell densities, or VCD, plotted against time for the CHOK1 cell line and the C1s^-/-^ CHOK1 2.E7 cell line. VCD was counted prior to splitting cells every two or three days for ten passages.

Because previous studies [27,37,44] suggested that stable cell CHO-S lines (ThermoFisher) expressing gp120 tended to form clumps in suspension cultures, we initially surveyed gp120 expression in several other cell lines including DG44 and CHOK1. Of these cell lines, CHOK1 had better growth in our suspension culture system. We used CRISPR/Cas9 gene editing to knockout the C1s protease in a suspension-adapted CHOK1 cell line. The C1s gene contains 11 exons, with exon 11 coding for the serine protease domain (Fig 2A) [45]. In a previous study, we screened potential guide RNAs targeting various exons within the C1s gene for knockout efficiency and found one that efficiently knocked out C1s proteolytic activity in the CHO-S cell line [27]. In this study, we used the same guide RNA targeting exon 11 to inactivate the C1s gene in CHOK1 cells. The procedure for creating the C1s^-/-^ CHOK1 cell line is described in detail in the Methods and Materials section. Using this strategy, 32 clones transfected with the CRISPR/Cas 9 guide RNA were analyzed by immunoblot. The supernatants of these clones were screened for the ability to cleave exogenous purified rgp120 and of these clones, three appeared to lack C1s protease activity (Fig 2B). The C1s gene from these three clones were PCR-amplified from genomic DNA extracted from CHO cells and all three were verified as successful knockouts by Sanger sequencing. To select the highest producing clone, clones 2.E7, 2.E9 and 3.E8 were transiently transfected with BaL-rgp120 (Fig 2C). Of these, clone 2.E7 was selected for use as the C1s^-/-^ CHOK1 cell line. The C1s gene has two copies in CHO cells [27]. A different indel was observed in each copy: an insertion of one thymine in one gene and an insertion of two thymines in the other (Fig 2D and Table 4). To verify that genetic inactivation of the C1s gene prevented proteolysis of gp120, we expressed the genes encoding three different clade B gp120s, EN2, EN6 and EN7, in the C1s^-/-^ CHOK1 2.E7 cell line and compared their sensitivity to proteolysis with the same proteins expressed in the normal CHOK1 cell line possessing an intact C1s gene (Fig 3A). As expected, inactivation of the C1s gene prevented proteolysis of all three proteins.

**Table 4:** C1s allele sequences for C1s^-/-^ CHOK1 clone 2.E7.

Wildtype C1s sequence	GTTGACAGCCGCTCATG↓TTGTGG	
Mutation in Allele 1	GTTGACAGCCGCTCATG↓TTTTGTGG	Inserted TT
Mutation in Allele 2	GTTGACAGCCGCTCATG↓TTGTGG	Deleted T

The sequence around the indel site was PCR amplified and cloned into a plasmid in order to sequence the two different mutations for each allele. The sequences of the two mutations observed at the expected indel site resulting in knockout of C1s in the CHOK1 cell line. The arrow indicates where the expected indel should occur, or three nucleotides before the protospacer adjacent motif (PAM). In this case, the PAM site is the last three nucleotides, TGG. Letters in bold indicate an insertion while letters with strikethrough indicate deletion.

Next, we examined the genetic stability of the C1s^-/-^ CHOK1 2.E7 cell line. Viable cell densities were recorded over ten passages every two to three days (Fig 3B). The C1s^-/-^ CHOK1 2.E7 cell line had a doubling time of 24.5 hours which was comparable to, and slightly better than the parental, suspension-adapted CHOK1 cell line, which had a doubling time of 26.6 hours. The shorter doubling time of the C1s^-/-^ CHOK1 2.E7 cell line is likely due to the clonal selection process for the C1s^-/-^ CHOK1 2.E7 line compared to the heterogenous CHOK1 cell line. Thus, the C1s^-/-^ CHOK1 2.E7 cell line is a robust cell line and inactivation of the C1s gene did not have deleterious effects on cell growth.

### Creation of the C1s^-/-^ MGAT1^-^ CHOK1 cell line

Previous studies have shown that many broadly neutralizing monoclonal antibodies to HIV recognize glycan dependent epitopes in gp120 [32,33,46,47]. Moreover, these studies showed that efficient binding of these antibodies required the mannose-5 form of N-linked glycosylation rather than the normal mature sialic acid containing form of N-linked glycosylation. The shorter, high-mannose glycans more closely resemble the type of glycosylation on authentic HIV virions which imparts enhanced binding of bN-mAbs [37,48,49,50]. Previously, we showed that gene editing could be used to inactivate the MGAT1^-^ gene, a critical intermediate in the N-linked glycosylation pathway [37]. Inactivation of this gene resulted in enrichment for mannose 5 and earlier intermediates in the N-linked glycosylation pathway. Therefore, it was of considerable interest to create a CHOK1 cell line deficient in both the MGAT1 and C1s genes for the production of clade B gp120s. For this purpose, we used CRISPR/Cas9 to inactivate the C1s gene in an MGAT1^-^ CHOK1 cell line made in a similar manner to the MGAT1^-^ CHO-S cell line described in a previous paper from our lab [37]. The resulting C1s^-/-^ MGAT1^-^ CHOK1 1.A1 cell line was used to express several clade B rgp120s.

**Fig 4:**
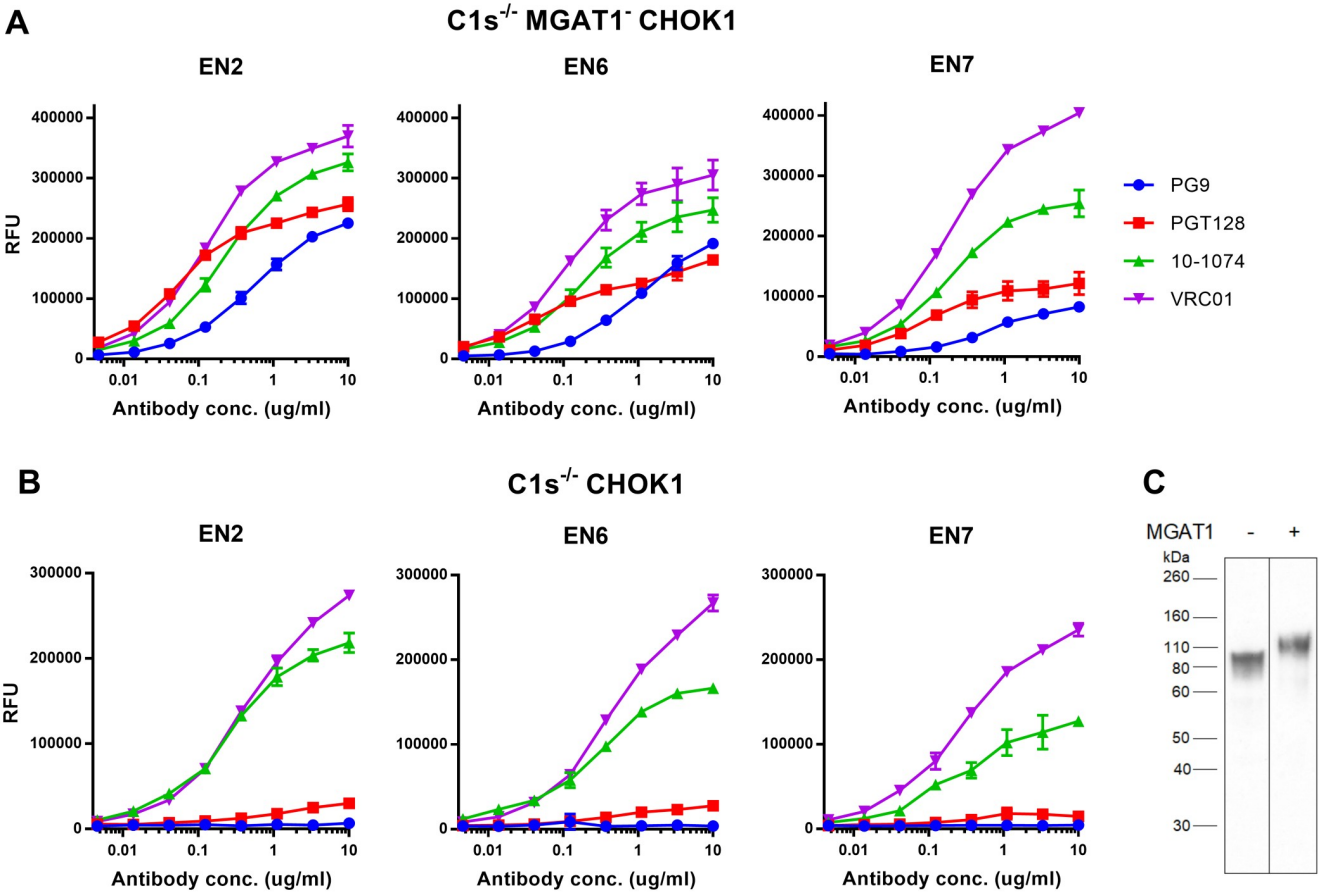
Enhanced binding of glycan-dependent bN-mAbs to clade B rgp120s expressed in cell lines deficient in C1s and MGAT1. A. Plasmids encoding three different gp120s (EN2, EN6, and EN7) were transfected into the C1s^-/-^ MGAT1^-^ CHOK1 1.A1. Diluted cell culture supernatants were collected after 4 days, captured with the 34.1 mAb for a capture fluorescence immunoassay (FIA) and assayed against a panel of bN-mAbs. B. The same EN2, EN6 and EN7 rgp120 monomers were transiently expressed in the C1s^-/-^ CHOK1 2.E7 cell line to show differences in antigenicity due to knockout of MGAT1 when compared to the C1s^-/-^ MGAT1^-^ CHOK1 1.A1 cell line. All assays were performed in triplicate. C. EN2 was expressed in the C1s^-/-^ MGAT1^-^ CHOK1 1.A1 cell line, indicated with a (-), and the C1s^-/-^ CHOK1 2.E7 cell line, indicated with a (+). Growth-conditioned cell supernatant was immunoblotted with an anti-rgp120 goat polyclonal antibody.

We tested the ability of the EN2, EN6 and EN7 rgp120s to bind a panel of bN-mAbs representative of major glycan dependent epitopes. We have previously shown that gp120s expressed in the MGAT1- CHO-S cell line displayed properly truncated oligomannose glycans [37]. EN2 rgp120 expressed in the C1s^-/-^ MGAT1^-^ CHOK1 1.A1 cell line had a lower molecular mass of about 120 kDa compared to EN2 gp120 expressed in the C1s^-/-^ CHOK1 2.E7 cell lines with a mass of 95 kDa (Fig 4C). This was expected since the loss of multiple sialic acid residues per glycan has been shown to cause a visible change in molecular weight [37,51]. We found that Envs produced in the C1s^-/-^ MGAT1^-^ CHOK1 1.A1 cell line (Fig 4A) displayed substantially greater binding to the glycan-dependent bN-mAbs, PG9 and PGT128 [34,46], compared to the same Envs expressed in the C1s^-/-^ CHOK1 2.E7 cell line (Fig 4B). Binding of the glycan dependent 10–1074 bN-mAb and the glycan independent VRC01 bN-mAb remained the same in both cell lines [35,52].

**Fig 5:**
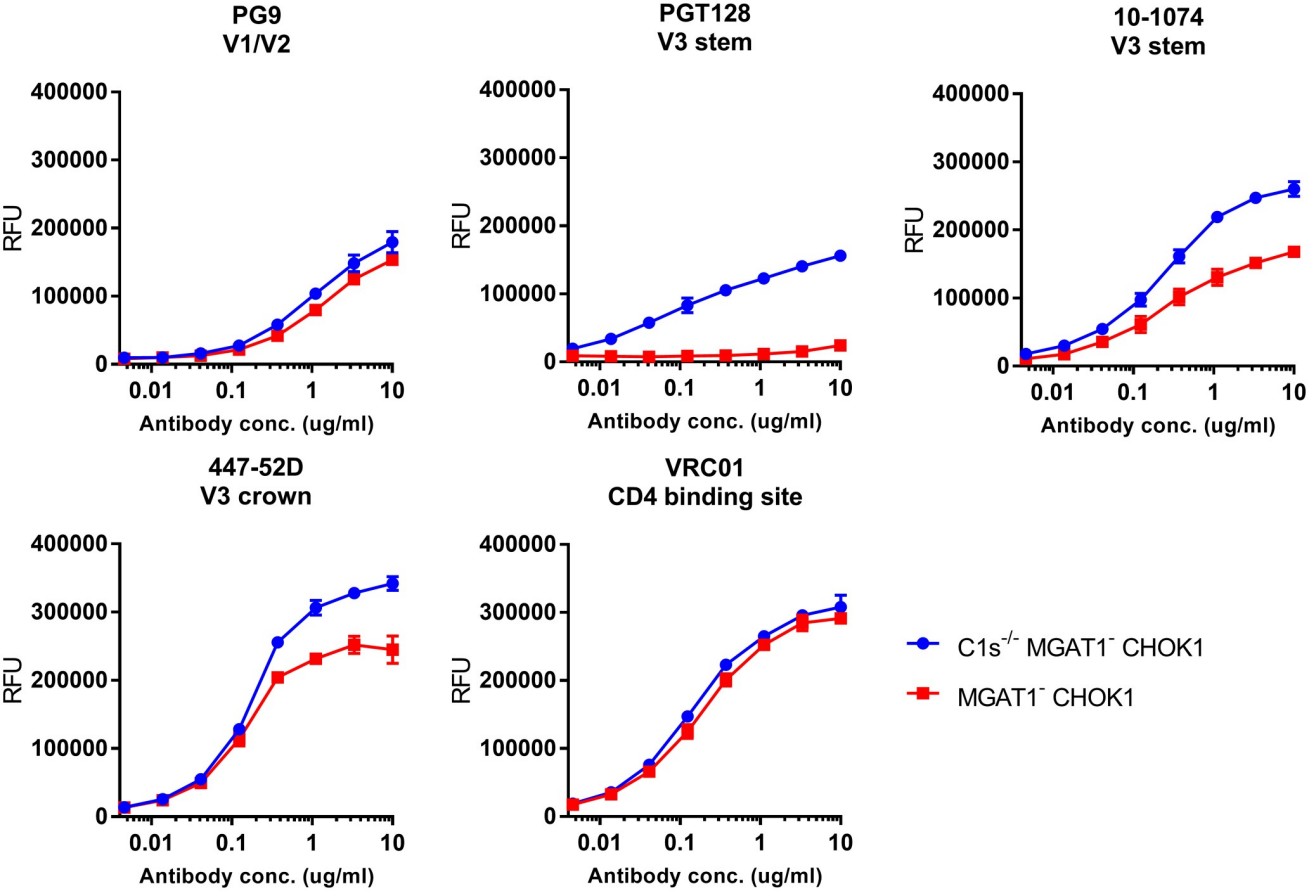
Effect of proteolysis by C1s on the binding of neutralizing monoclonal antibodies and broadly neutralizing monoclonal antibodies to EN6 rgp120. Plasmids encoding EN6 rgp120 were transiently expressed in the C1s^-/-^ MGAT1^-^ CHOK1 1.A1 and MGAT1^-^ CHOK1 cell lines. Growth-conditioned cell culture supernatants were diluted for a 34.1 mAb capture FIA. All assays were performed in triplicate. Indicated under the antibody names are the epitope recognized by the respective antibodies.

To explore the effect of proteolysis by the C1s protease on antigenicity of rgp120, we compared EN6 rgp120 expressed in the C1s^-/-^ MGAT1^-^ CHOK1 1.A1 cell line (not proteolyzed EN6) and MGAT1^-^ CHOK1 cell line (proteolyzed EN6) (Fig 5). As expected, we found that the binding of EN6 rgp120 to PG9 and VRC01 was not affected by the C1s protease inactivation as these bN-mAbs recognize the V1/V2 domain and CD4 binding site, respectively. However, binding of proteolyzed EN6 to PGT128, which recognizes the V3 stem, was significantly decreased as is consistent with the presence of the C1s site. Similarly, binding of proteolyzed EN6 to bN-mAb 10–1074, which recognizes the V3 stem, and mAb 447-52D, which recognizes the V3 crown, was also diminished [31]. Thus, expression of Envs in the C1s^-/-^ MGAT1^-^ CHOK1 1.A1 cell line prevented proteolysis of the rgp120s at the V3 loop and promoted binding to glycan-dependent and V3 bN-mAbs.

### Partial proteolysis of Factor VIII thrombin cleavage sites in gp120-Factor VIII chimeric constructs

**Fig 6:**
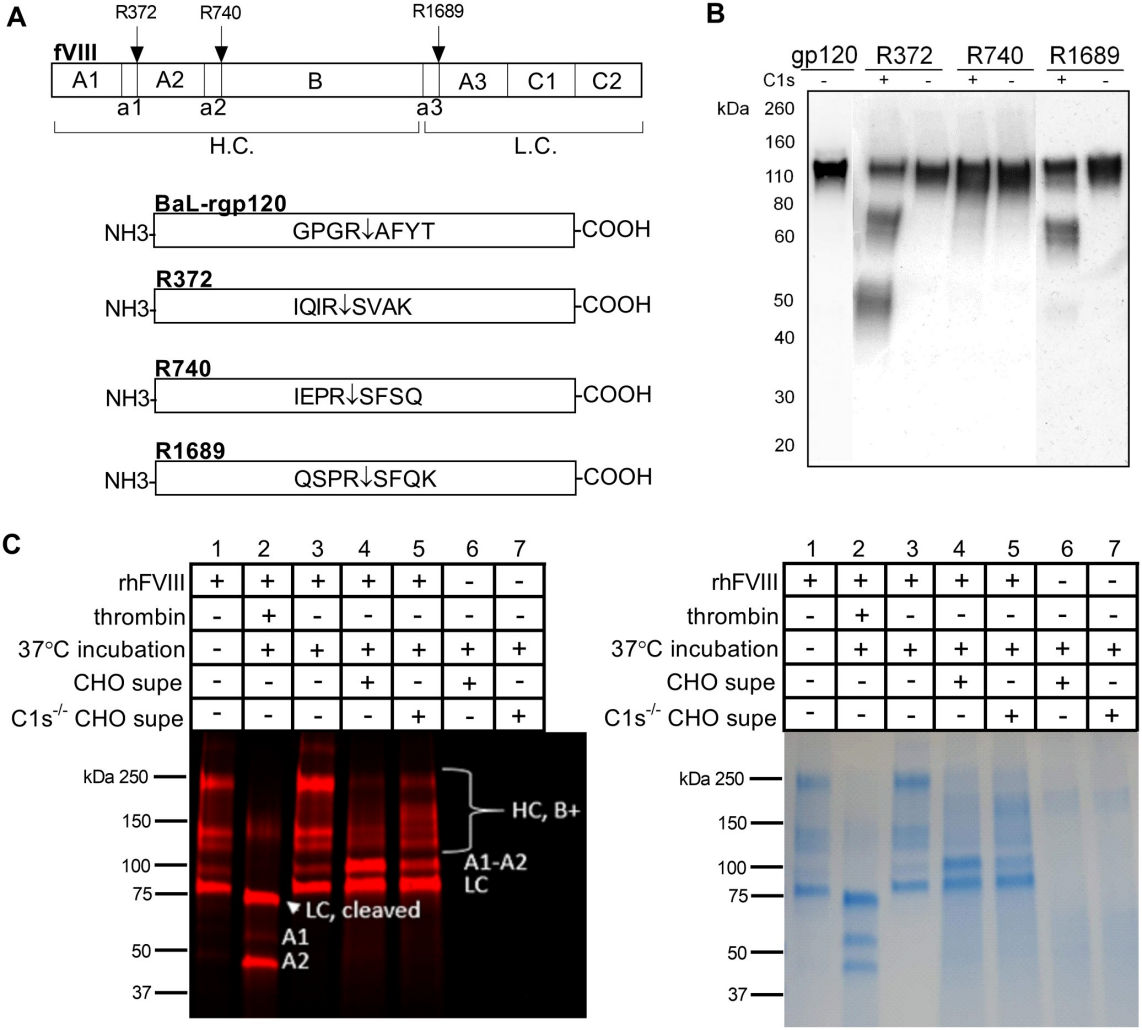
Limited proteolysis of recombinant human Factor VIII by CHO cell-derived C1s. A. The FVIII gene is shown with the three thrombin cleavage sites, R372, R740 and R1689. The heavy chain and light chain are labeled H.C. and L.C. Chimeric gp120-FVIII constructs were created by replacing the C1s cleavage site in the V3 crown of gp120 with thrombin cleavage sites from Factor VIII. The three thrombin cleavage sites on human Factor VIII were cloned into BaL-rgp120 to replace the GPGR↓AFYT sequence. Three cloned constructs were created with each of the three thrombin cleavage sites. B. Transient expression and cleavage of the chimeric gp120-Factor VIII constructs R372, R740 and R1689 in either the CHOK1 cell line or the C1s^-/-^ CHOK1 2.E7 cell line. Chimeric constructs expressed in the CHOK1 cell line are indicated with (+) and those expressed in the C1s^-/-^ CHOK1 2.E7 cell line are indicated with (-). Cell culture supernatant was assayed by immunoblot, and samples were all run reduced with DTT. C. Recombinant human Factor VIII (rhFVIII), also known as Advate, was labeled with DyLight650 and tested for proteolysis following treatment with CHO cell and C1s^-/-^ CHOK1 2.E7 cell supernatants. Shown are analyses of FVIII fragments by SDS-PAGE. Thrombin was incubated with rhFVIII as a positive control to observe the expected protein fragments after proteolysis at R372, R740 and R1689. Factor VIII fragments are labeled on the immunoblot as HC (heavy chain), LC (light chain), and the A1 and A2 domains.

Next, we wanted to explore the substrate specificity of C1s by measuring its effect on other recombinant proteins susceptible to proteolysis. For these studies, we selected human factor VIII (FVIII). Human FVIII is a large protein of 2332 amino acids, is part of the coagulation pathway, and is necessary for the formation of fibrin found in blood clots [53]. It undergoes both maturational cleavages mediated at a furin-cleavage site and by thrombin as well as artifactual proteolysis mediated by an unknown protease when expressed in CHO cells [53–55]. FVIII is activated through cleavage by thrombin at R372 (Arg372), R740 and R1689 (Fig 6A) [56]. Similar to rgp120, artifactual proteolysis of FVIII is mediated by an endogenous CHO protease. This proteolysis occurs at FVIII’s three thrombin cleavage sites [55]. Both thrombin and C1s are serine proteases, and since thrombin is able to cleave the GPGRAF sequence in gp120, we wanted to examine the possibility that C1s was the endogenous protease responsible for artifactual proteolysis of recombinant Factor VIII when expressed in CHO cells.

In a pilot study, we created chimeric gp120-FVIII constructs where the GPGR motif in the V3 loop was replaced by each of the FVIII cleavage sites to observe whether the FVIII thrombin cleavage sites were sensitive to proteolysis by C1s. The V3 sequence, GPGR/AFYT, was replaced with each of the three thrombin cleavage sites, R372 (IQIR/SVAK), R740 (IEPR/SFSQ), and R1689 (QSPR/SFQK), such that these sequences would be accessible to C1s and could be assayed by the same immunoblot methodology used to study the proteolysis of rgp120 (Fig 6A). The chimeric rgp120-Factor VIII constructs were transiently transfected in the CHOK1 and C1s^-/-^ CHOK1 2.E7 cell line. We found that C1s, secreted by the normal CHOK1 cells, was able to cleave the chimeric rgp120s. The R372 and R1689 thrombin cleavage sites in the gp120-Factor VIII chimeric constructs were cleaved by CHO C1s, but the R740 thrombin cleavage site was resistant to cleavage (Fig 6B).

### Resistance of recombinant human Factor VIII to proteolysis by CHO C1s

While the chimeric constructs provided some information on the substrate specificity of C1s, we were interested in treating Factor VIII in its native structure with C1s to observe its susceptibility to C1s-mediated proteolysis. In these studies, we utilized Advate, a recombinant FVIII therapeutic protein. FVIII was incubated with growth-conditioned supernatants from CHOK1 cells and C1s^-/-^ CHOK1 2.E7 cells to determine whether proteolysis of FVIII occurred after treatment with CHOK1 cell supernatants and whether supernatants from cells lacking C1s prevented the proteolysis. FVIII is composed of a glycosylated 200 kDa heavy chain and an 80 kDa light chain, held together by a divalent metal ion (Fig 6A) [57]. In lane 2, Advate was cleaved by thrombin to show the expected cleavage products (Fig 6C). The heavy chain was cleaved in two places resulting in three fragments: the A1 domain, the A2 domain and the B domain. The 80 kDa light chain was cleaved to a 73 kDa fragment. In lanes 3 and 4, proteolysis of the heavy chain was observed at the furin cleavage sites at R1313 and R1648 for Advate treated with supernatants from both the C1s-containing and C1s-deficient cell lines. This indicated the presence of a furin-like protease secreted from CHO cells which was not C1s [58]. Slightly less proteolysis of the heavy chain for the Advate + C1s^-/-^ CHOK1 2.E7 supernatant than with Advate + CHOK1 supernatant was observed, indicating that some proteolysis was caused by C1s. The expected FVIII fragments of the cleaved light chain at 73 kDa, the A1 domain at 50 kDa and the A2 domain at 43 kDa after cleavage by thrombin were not observed when FVIII was incubated with CHO supernatant, suggesting that CHO C1s was not involved in the proteolysis of FVIII despite the fact that the R372 and R1689 sites was susceptible to cleavage in the gp120-FVIII chimeric constructs. The accessibility of the thrombin cleavage sites is different in Advate, the recombinant human Factor VIII protein, compared to those in the chimeric gp120-FVIII constructs we created. In the gp120-FVIII constructs, the thrombin cleavage sites were placed in the V3 loop, a long, extended loop of approximately 35 residues. Normally, the V3 loop extends past the core of gp120 to bind to the co-receptor on T-cells. However, this also makes the loop accessible to proteolysis. Conversely, the thrombin cleavage sites on human Factor VIII only serve to activate the protein. These cleavage sites might not be readily proteolyzed by C1s due to tertiary and quarternary structure imparted by the multiple, globular domains of Factor VIII. In sum, we did not observe the proteolysis of recombinant Factor VIII when expressed in CHO cells as described by Kaufmann et al. [55].

### The substrate specificity of CHO C1s

**Fig 7:**
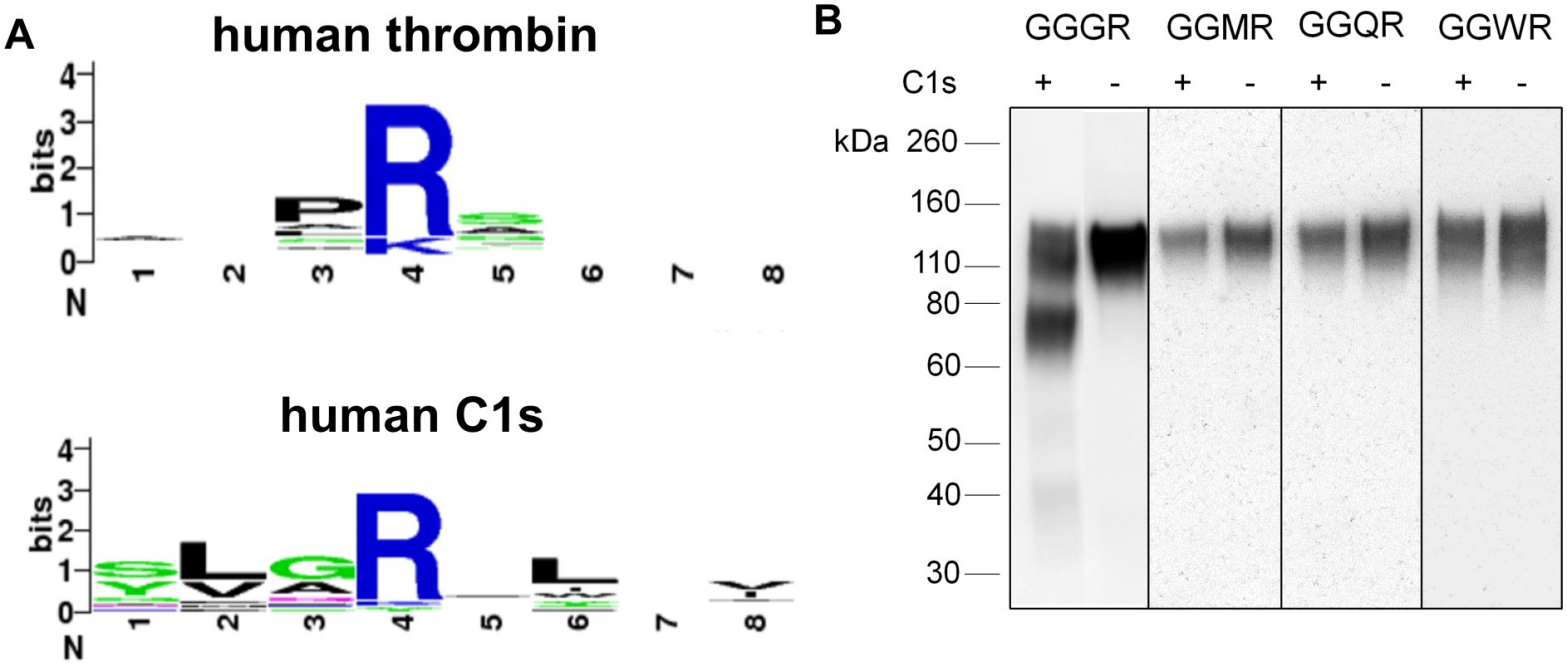
Determining the substrate specificity of C1s. A. The cleavage site sequence logos for human thrombin and human C1s are graphed based on data from the MEROPS database [62]. Positions 1 through 8 represent positions P4-P3-P2-P1-P1’-P2’-P3’-P4’ as per conventional nomenclature for substrates of proteases. B. Expression of gp120 variants with amino acid substitutions in the V3 crown sequence. GGSR, GGMR, GGQR and GGWR were substituted for GPGR in the V3 crown of BaL-rgp120. BaL-rgp120 was transiently expressed in the CHOK1 cell line and C1s^-/-^ CHO 2.E7 cell line. Mutagenesis constructs expressed in the CHOK1 cell line are indicated with (+) and those expressed in the C1s^-/-^ CHOK1 2.E7 cell line are indicated with (-). Cell culture supernatant was assayed by immunoblot, and all samples were reduced with DTT.

While the substrate specificity of thrombin has been well-studied, the substrate specificity of C1s produced in CHO cells has not been determined. Here, we compare the substrate specificity of CHO C1s to human thrombin to observe differences in the amino acids they favor and the substrates they will cleave. Because human C1s shares a 77% amino acid sequence identity with CHO C1s and has a known substrate specificity and solved crystal structure, we used human C1s to determine the specificity of CHO C1s [59–61]. Human C1s prefers substrates with small amino acids such as glycine and alanine in the P2 position, as shown in the cleavage site sequence logo from the MEROPS peptidase database (Fig 7A) [62]. The common nomenclature for protease substrates, P4-P3-P2-P1-P1’-P2’-P3’-P4’, is used where P1 is the residue immediately before the scissile bond. CHO C1s also shows a similar substrate specificity for small amino acids in the P2 position based on experiments with HIV Env and the physiological relevant substrates of C1s, C2 and C4 (Table 5). CHO C1s cleaves HIV Envs with the GPGR motif as well as the complement component C2 protein, both of which have glycine in the P2 position. CHO C1s also cleaves complement component C4 which has an alanine in the P2 position. Thus, CHO C1s efficiently cleaves substrates with a small amino acid in the P2 position.

**Table 5:** Known substrates of CHO C1s and the substrates tested for proteolysis by CHO C1s.

Substrate	P_4_	P_3_	P_2_	P_1_	P_1_'	P_2_'	P_3_'	P_4_'	Cleaved by CHO C1s	Side Chain Type
Clade B BaL gp120	Gly	Pro	**Gly**	Arg	Ala	Phe	Tyr	Thr	**+**	small
complement component C2	Asn	Leu	**Gly**	Arg	Arg	Ile	Gln	Ile	**+**	small
complement component C4	Gly	Leu	**Ala**	Arg	Ala	Gln	Glu	Val	**+**	small
Factor VIII R372	Ile	Gln	**Ile**	Arg	Ser	Val	Ala	Lys	**+**	medium
Factor VIII R740	Ile	Glu	**Pro**	Arg	Ser	Phe	Ser	Gln	**-**	bulky
Factor VIII R1689	Gln	Ser	**Pro**	Arg	Ser	Phe	Gln	Lys	**+**	bulky
GGGR-GGGG	Gly	Gly	**Gly**	Arg	Gly	Gly	Gly	Gly	**+**	small
GGMR-GGGG	Gly	Gly	**Met**	Arg	Gly	Gly	Gly	Gly	**-**	long
GGQR-GGGG	Gly	Gly	**Gln**	Arg	Gly	Gly	Gly	Gly	**-**	long
GGWR-GGGG	Gly	Gly	**Trp**	Arg	Gly	Gly	Gly	Gly	**-**	long, bulky

Shown are the amino acids in the P4-P4’ positions of the substrates, the susceptibility to proteolysis by CHO C1s, and a descriptor for the amino acid in the P2 position.

Testing for the proteolysis of the thrombin cleavage sites on Factor VIII further revealed the nuanced substrate specificity of CHO C1s. As discussed previously, CHO C1s recognized two out of three thrombin cleavage sites, R372 and R1689, in the gp120-Factor VIII chimeric constructs (Fig 7C). R372 has the sequence IQIR/SVAK and has an isoleucine in the P2 position. This sequence was recognized by CHO C1s due to the similarity between isoleucine and alanine, which have short, aliphatic side chains. R1689 has the sequence QSPR/SFQK with a serine in the P3 and a proline in the P2 position. Based on the premise that C1s prefers small amino acids in the P2 position, it was surprising to see that CHO C1s recognized and cleaved this particular sequence due to the larger and more bulky proline in the P2 position. It is possible that the presence of the serine, a small amino acid, in the P3 position allowed C1 to accommodate larger amino acids in the P2 position. The third thrombin cleavage site, R740, which has the sequence IEPR/SFSQ with a glutamine in the P3 position and a proline in the P2 position, was not cleaved by CHO C1s. The long-chained, negatively charged glutamine in combination with the proline made this substrate inaccessible to CHO C1s.

Mutagenesis of the P2 position of gp120-Factor VIII constructs was done to determine the extent by which proteolysis is controlled by the residue in the P2 position. The P4 position was kept as arginine, while all other residues in the P4-P4’ positions other than P2 were mutated to glycine, to create a GGXRGGGG sequence (Fig 7B). Glycine (G), methionine (M), glutamine (Q) and tryptophan (W) were mutated into the P2 position based on peptide microarray substrate specificity data for human C1s from Gosalia et al [63]. The peptide microarray data showed that C1s would not cleave substrates with methionine, glutamine or tryptophan in the P2 position, but could cleave substrates with glycine in the P2 position. gp120-Factor VIII constructs with serine, methionine, glutamine and tryptophan in the P2 position were transiently expressed in the CHOK1 and C1s^-/-^ CHOK1 2.E7 cell lines to determine whether CHO C1s could cleave these substrates. The construct with glycine in the P2 position was cleaved, but the constructs with methionine, glutamine and tryptophan were not cleaved. This data further supported the conclusion that CHO C1s could cleave substrates with small amino acids including glycine and alanine in the P2 position but would be obstructed by substrates with a larger residue.

Thrombin has been shown to cleave substrates including clade B gp120s, coagulation factor proteins, and fibronectin and can recognize substrates with not only proline in the P2 positions, but also hydrophobic and non-aromatic amino acids including glycine, alanine, valine, leucine, isoleucine and glutamine based on data from the MEROPS peptidase database (Fig 7A) [22,64]. However, we have shown that CHO C1s has a more limited specificity as it cannot cleave the R740 thrombin cleavage site in our gp120-Factor VIII chimeric constructs. Additionally, we showed that CHO C1s is specifically hindered by the residue in the P2 position, as it cannot cleave substrates with methionine, glutamine or tryptophan in the P2 position. These experiments have shown that CHO C1s has a narrower specificity than thrombin but in certain cases, can accommodate amino acids such as isoleucine or proline in the P2 position when flexibility of the substrate is imparted by a smaller residue in the P3 position.

### Structural analysis of the CHO C1s substrate binding site

**Fig 8:**
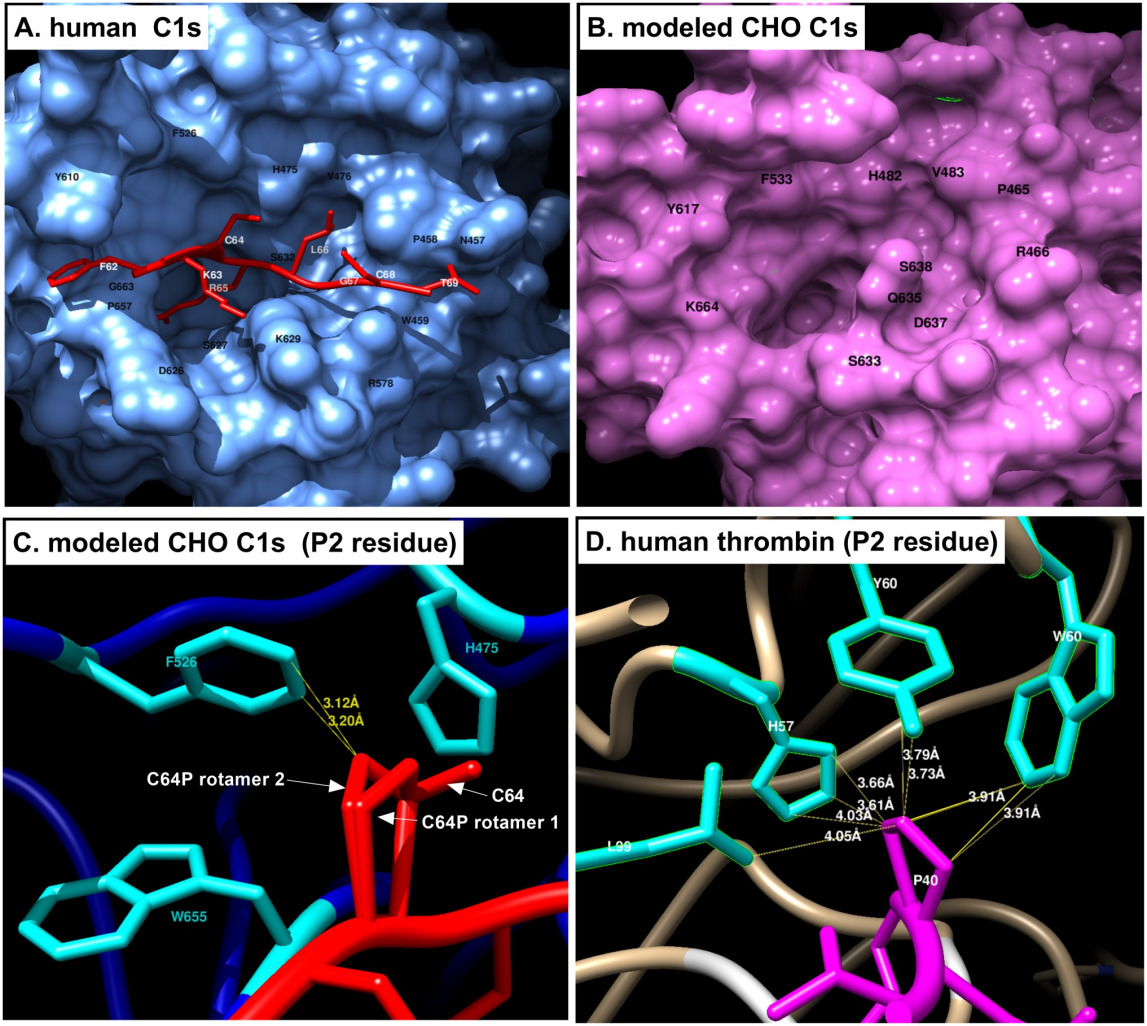
Homology modeling of the structure of the CHO C1s active site. A. The crystal structure of human C1s with the substrate gigastasin (PDB 5UBM) was visualized using Chimera. The P4-P4’ residues of gigastasin are in red, while the protease is in blue. Residues on the protease that interact with the substrate are labeled in black. The P4-P4’ residues of the substrate are labeled in white. B. A homology model of CHO C1s was created with SWISS-model using the sequence of CHO C1s and the crystal structure of human C1s as a template. In black are the residues of CHO C1s that likely interact with the substrate based on the homology to human C1s and predicted van der Waals interactions. C. A zoomed in view of Cysteine64 (C64) in the P2 position of gigastasin (red) with human C1s (blue). Residues within 5 Angstroms of C64 are in cyan. C64 was computationally mutated into a proline. Shown are two possible rotamers using the Dunbrack 2010 rotamer library in Chimera. Parameters for clashing were as follows: atoms with VDW overlap > = 0.4 angstroms, subtract 0.2 from overlap for potentially H-bonding pairs. Distances in yellow are those involved in clashes. D. A zoomed in view of human thrombin (tan) with its substrate, protein C (magenta) (PDB 4D7Y). Distances between Proline40 of the substrate were identified with default contact parameters.

To understand how the structure of CHO C1s determines its substrate specificity, we studied the previously solved crystal structure of human C1s in complex with the substrate, gigastasin (PDB 5UBM) (Fig 8A) [61]. The residues Y610 and F526 interact with the P4 residue F62 to form a hydrophobic box, a feature also seen in coagulation factor proteases that favors a bulky, hydrophobic residue in the P4 position [65]. The P3 residue K63 protrudes out and away from the protease. The P2 residue C64 is small and restricted by the bulky F526 and H475. The P1 residue R65 is in the S1 pocket in which D626 forms the bottom and G663 and S627 form the sides. The catalytic serine S632 is situated adjacent to the S2 pocket. The charged K629 potentially obstructs unsuitably sized substrates from entering the substrate binding site. The C-terminal side of the scissile bond is moderately restricted by W476 and the more distal residues P458, W457, W459 and R578.

A model of CHO C1s was made using SWISS-model using the crystal structure of human C1s as a template (PDB 1ELV) (Fig 8B). This structure was used in the molecular replacement for the above crystal structure of human C1s with gigastasin. The homology model of CHO C1s is similarly structured to human C1s with minor differences. The hydrophobic box, consisting of Y617 and F533, are still conserved and the positions of the F533 and H482 are similar to the corresponding residues in human C1s, limiting the size of the residue in the P2 position. The S1 pocket is clearly visible and is bounded by D637 at the bottom and S638 at the side. The catalytic serine is present in approximately the same place, adjacent to the S1 pockets. A glutamine, Q535, now blocks the entrance to the P1 pocket instead of the lysine in human C1s. The C-terminal side of the scissile bond remain relatively unchanged.

A closer look into the interactions between the P2 residue of the substrate and surrounding residues of the protease explains the ability of thrombin, but not C1s to cleave residues with a proline in the P2 position. Mutating C64, the P2 residue of gigastasin, to proline shows potential clashing (Fig 8C). One of the two rotamer forms of proline that tilts towards F526 clashes with it and has a van der Waals overlap between 0.4 and 0.6 angstroms. A more severe clash would have a van der Waals overlap greater than 0.6 angstroms. This moderate overlap can sometimes be resolved by a smaller residue such as a serine in the P3 position to accommodate larger residues in the P2 position as was observed with the Factor VIII R1689 cleavage site (Fig 6B and Table 5). When substrate protein C is complexed with thrombin, the proline, P40, in the P2 position does not clash with the surrounding residues and distances between the proline and the surrounding residues are greater than those in C1s (Fig 8D).

## Discussion

Although CHO cells are widely used for the production of most protein therapeutics, limited proteolysis, or clipping, is a common problem in large scale production of recombinant proteins. Clipping can be cell line-specific or protein-specific, and there are multiple CHO cell lines with varying chromosomal structures and genome sequences that are still in the process of being characterized for protease activity [66]. The assembly of the CHO cell genome in 2011 and the discovery of gene-editing technology has provided insights into the best ways to engineer CHO cells to optimize the expression of recombinant proteins [67]. The zinc-finger matriptase knockout cell line demonstrates the ongoing need for protease-inactivated cell lines [8]. A glutamine synthetase knockout cell line has been shown to enhance the clone selection process and increase productivity, while a transcriptomics approach to CHO cell engineering led to knockout of the repressors of STAT1, a transcription factor that elicits a cytokine response, that enhanced resistance of CHO cells to viral contamination [8,68–70]. Recent papers have identified Cathepsin D as a protease detrimental to the expression of recombinant proteins from CHO cells and used zinc-finger nucleases to knockout matriptase to create a protease-knockout cell line [7,8]. A total of four CRISPR/Cas9 gene-edited cell lines have been developed by our lab for the production of recombinant proteins (Table 6). Here, we describe the identity of another protease (C1s) that results in the degradation of another recombinant protein, specifically clade B, HIV Env proteins used as vaccine immunogens. The creation of the C1s^-/-^ CHOK1 2.E7 cell line and the C1s^-/-^ MGAT1^-^ CHOK1 1.A1 cell line, and the analysis of C1s substrate specificity has created a practical solution to solve the proteolysis problem in the large-scale production of clade B, HIV vaccine immunogens and other recombinant proteins.

**Table 6:** CRISPR-Cas9 gene edited CHO cell lines for the expression of recombinant proteins.

Cell line	Journal Ref.
MGAT1^-^ CHO-S	Byrne et al. (2018)
C1s^-/-^ MGAT1^-^ CHO-S (stably expressing BaL-rgp120)	Li et al. (2019)
C1s^-/-^ CHOK1 2.E7	shown in this paper
C1s^-/-^ MGAT1^-^ CHOK1 1.A1	shown in this paper

These cell lines, except the C1s-/- MGAT1- CHO-S cell line, are available for transient or stable transfection of any recombinant protein. All cell lines were produced in our lab.

One of the existing solutions to prevent proteolysis of gp120s is mutating Arg315 of the GPGR motif to a Glutamine (Q). This mutation has been done for both gp120 monomers and SOSIP trimers found to undergo proteolysis, and highlights the applicability of the C1s-knockout cell lines [26,71]. While the R315Q mutation prevents the elicitation of antibodies dependent on arginine such as the neutralizing mAb 447-52D, it does not affect elicitation of antibodies to the V3 stem such as PGT128 or PGT121. At the same time, the R315Q mutation removes the GPGR motif, a highly-conserved motif in clade B strains and as a result, will prevent elicitation of potentially neutralizing antibodies. Lastly, changing the Env sequence may not be ideal in certain studies. For example, antibodies in human sera from elite neutralizers or controllers should be studied against the exact viral Env sequences. Otherwise, Envs to the Arg315 may be overlooked.

A number of groups are creating stable CHO cell lines expressing HIV envelope proteins for clinical trials. One such study hopes to evaluate different adjuvants with a multivalent vaccine using the RV144 subtype AE immunogen A244 and a subtype B immunogen, designated 6240 [26]. Other groups are expressing Env trimers in CHO cells such as gp140 trimer nanoparticles and SOSIP trimers [72–74]. In most cases, these vaccines were produced in normal CHO cells that incorporated complex, sialic acid-containing glycans that inhibited the binding of multiple, glycan-dependent bN-mAbs such as PG9, PGT121, and PGT128 [37,75]. These cell lines contained C1s which would degrade the final protein product, particularly when expressed at large scale for long fermentation runs. With these cell lines deficient in MGAT1 and C1s, we have shown that gene editing can solve both the problems of inappropriate glycosylation and limited proteolytic cleavage and that these cell lines can be utilized for production of HIV immunogens.

In these studies, we explored the differences between the substrate specificity of C1s and thrombin. Although thrombin has long been known to cleave rgp120 at the GPGR/AF sequence in the V3 region, it was interesting to discover that C1s also cleaved this sequence [22,23]. Although C1s did not cleave at the thrombin cleavage sites in the context of the FVIII protein, it was able to cleave two of three Factor VIII thrombin cleavage sites in the gp120 V3 domain. While C1s and thrombin share some similar substrate specificity, this demonstrates a conformational or accessibility requirement for C1s cleavage. These overlapping substrate specificities reflect the crosstalk observed between proteases and substrates in the coagulation and complement pathways [76]. Proteases in the coagulation system, thrombin, coagulation factors XIa, Xa, and IXa, and plasmin were found to cleave C3 and C5, substrates of the complement system [77]. Conversely, MASP1 and MASP2 of the lectin pathway in the complement system can cleave fibrinogen into fibrin and activate both Factor VIII and prothrombin [78]. It is thought that coagulation factor proteases evolved from complement proteases in vertebrates and that there is an ancestral group of protein domains from which complement proteases of arthropods and vertebrates originate [79]. Despite the fact that only thrombin can cleave substrates with bulkier proline in the P2 position of the substrate, this lineage shows that these serine proteases from seemingly two very different systems are evolutionarily, structurally and functionally similar.

With the knowledge of the substrate specificity of C1s, future issues of proteolysis with recombinant proteins can be avoided. The prevalence of proteases in the regulation of physiological processes suggests there will be continued recombinant expression of proteases and protease substrates. Of the approximately 20,000 proteins in the human proteome, 570 are proteases, or about 2.6% of the proteome [80,81]. Proteases regulate physiological processes such as endocrine hormone maturation, the blood coagulation cascade, the complement pathway, and virus maturation. Dysfunctional proteases or genetic variants of substrates that become resistant to proteolysis can result in fatal disease including hemophilia, lupus, etc [82]. The C1s^-/-^ CHOK1 2.E7 and C1s^-/-^ MGAT1^-^ CHOK1 1.A1 cell lines described in this report can be used for the expression of all recombinant proteins susceptible to proteolysis by C1s. The finding that CHO cell-derived C1s only cleaves substrates with a small amino acid prior to the arginine in the P1 position will help predict C1s cleavage sites in other proteins. Data mining of the MEROPS database for protein substrates with the sequences Gly-Arg or Ala-Arg in exposed loop regions may reveal a number of proteins that cannot be expressed in the normal CHO cell line. This information will guide how these proteins are expressed whether it be through the use of the C1s^-/-^ CHOK1 cell line, mutating away protease cleavage sites as was done for the CHO production of MN-rgp120 or use of an alternate cell line [26].

In summary, we have shown that gene editing can solve the problems of proteolysis and glycosylation heterogeneity encountered in the expression of HIV vaccine immunogens. In principle, this same approach can be employed to eliminate proteases and other glycan modifying enzymes that interfere with the manufacturing and production of other recombinant proteins of pharmaceutical interest.

## Supporting information

S1 Fig Physical characteristic of A244, EN3 rgp120s used in this study. The length in amino acids (AA) and number of potential N-linked glycosylation sites (PNGS) are listed for each of the HIV Env domains for the expressed Envs: A244, EN3_071.
(TIF)

S1 Raw images Raw images of gels and blots. (PDF)
