# Supplementary figures and images for: Gene editing in CHO cells to prevent proteolysis and enhance glycosylation: Production of HIV envelope proteins as vaccine immunogens
Source: PLoS One. 2020 May 29;15(5):e0233866. doi: 10.1371/journal.pone.0233866 (PMC7259603; doi:10.1371/journal.pone.0233866)

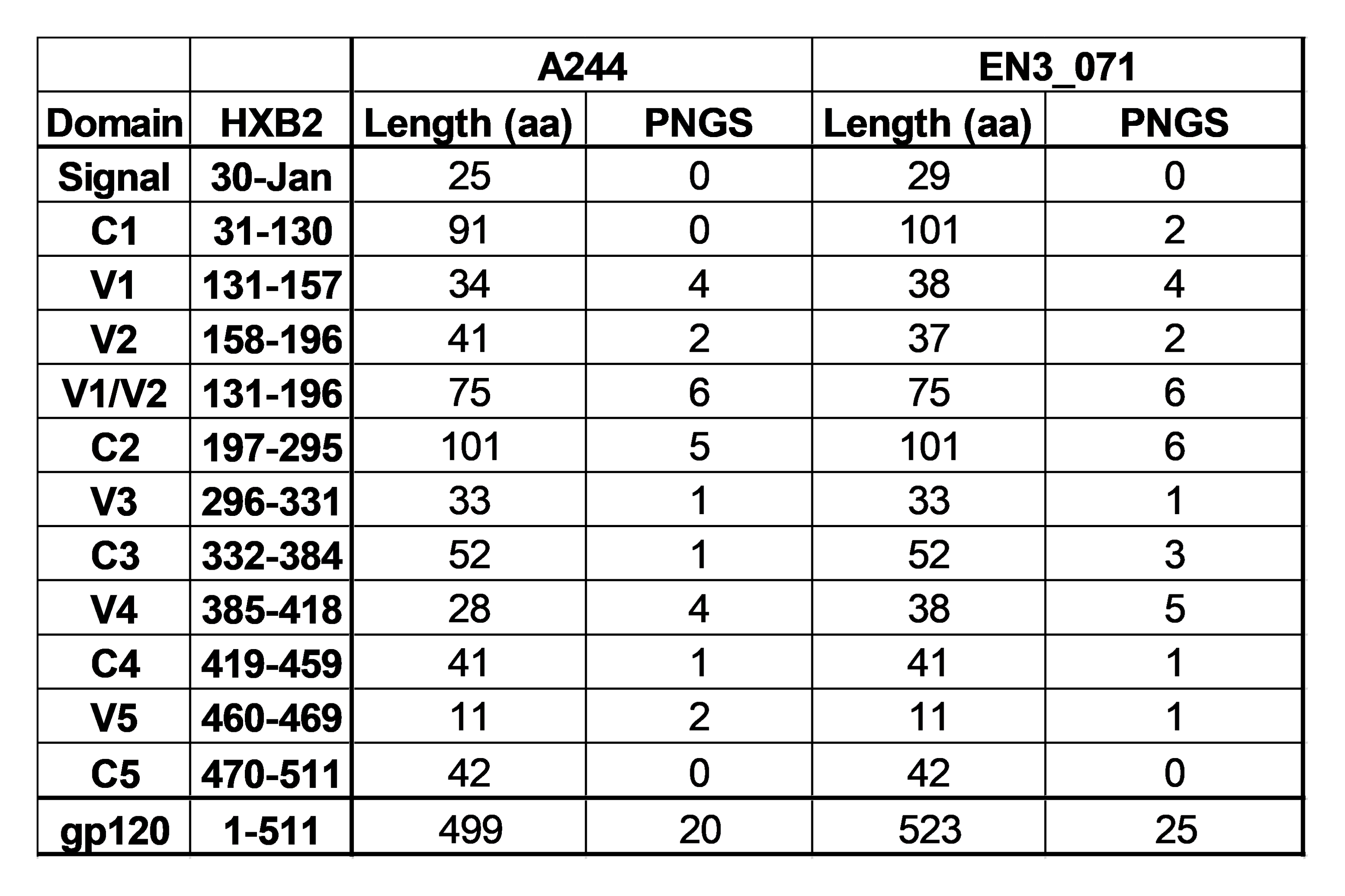

Supplement: S1 Fig — The length in amino acids (AA) and number of potential N-linked glycosylation sites (PNGS) are listed for each of the HIV Env domains for the expressed Envs: A244, EN3_071. (TIF) [file pone.0233866.s001.tif]
