# Supplementary material for: Gene editing in CHO cells to prevent proteolysis and enhance glycosylation: Production of HIV envelope proteins as vaccine immunogens
Source: PLoS One. 2020 May 29;15(5):e0233866. doi: 10.1371/journal.pone.0233866 (PMC7259603; doi:10.1371/journal.pone.0233866)
Supplement: S1 Raw images — (PDF) [file pone.0233866.s002.pdf]

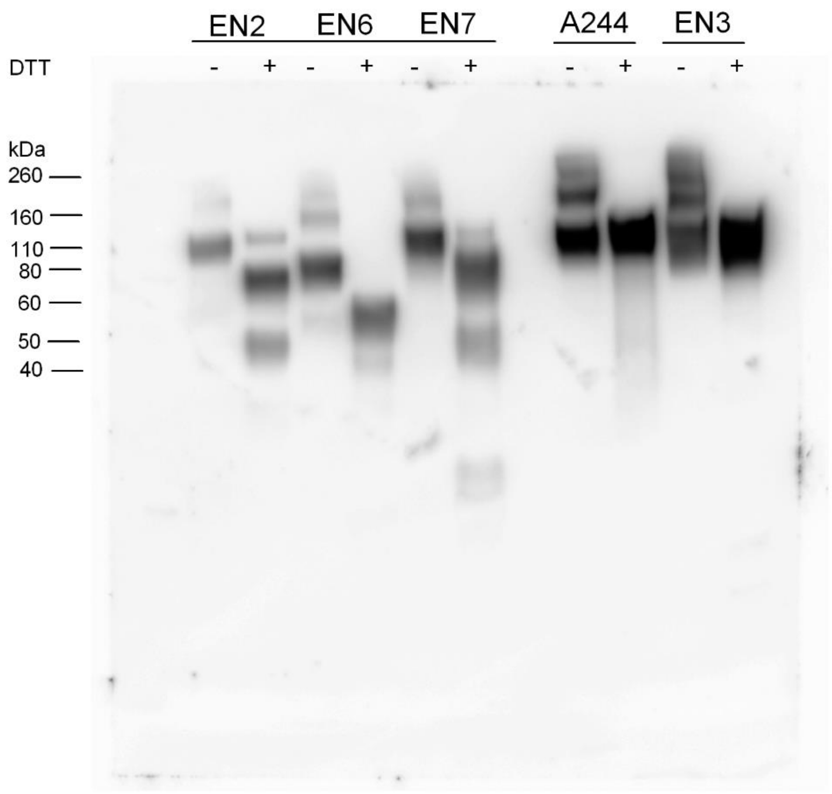

Figure 1. Western blot probed with anti-goat polyclonal conjugated to HRP.

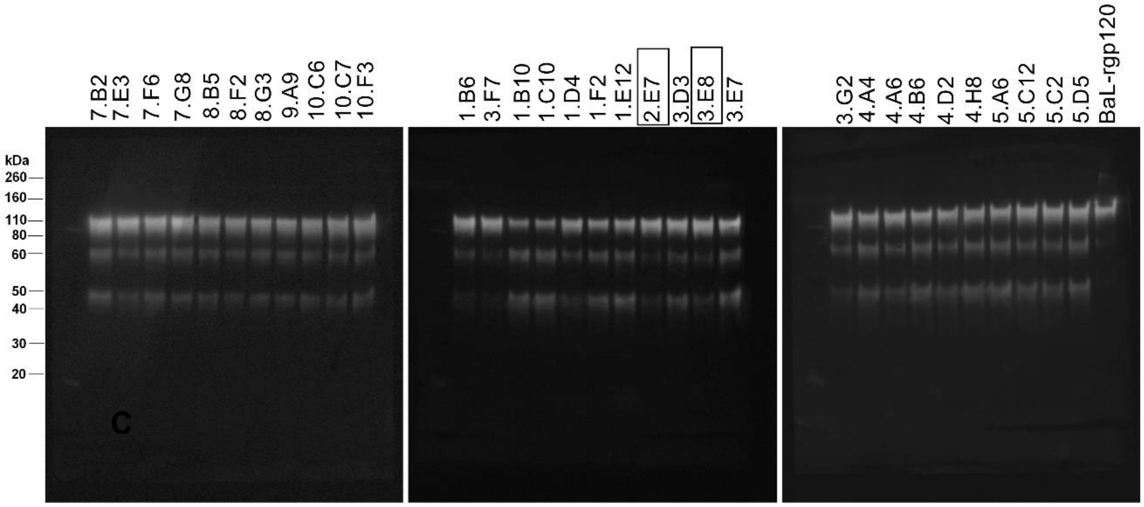

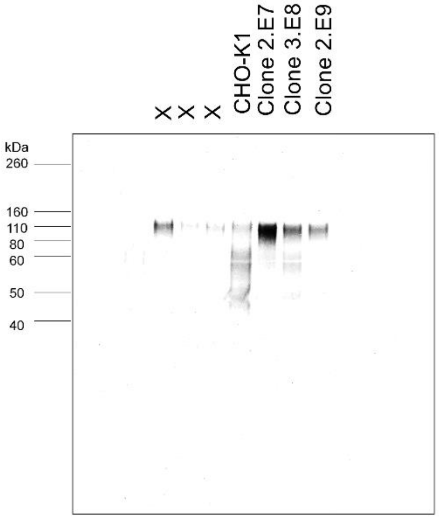

Figure 2B and 2C. Western blot probed with anti-goat polyclonal conjugated to HRP.

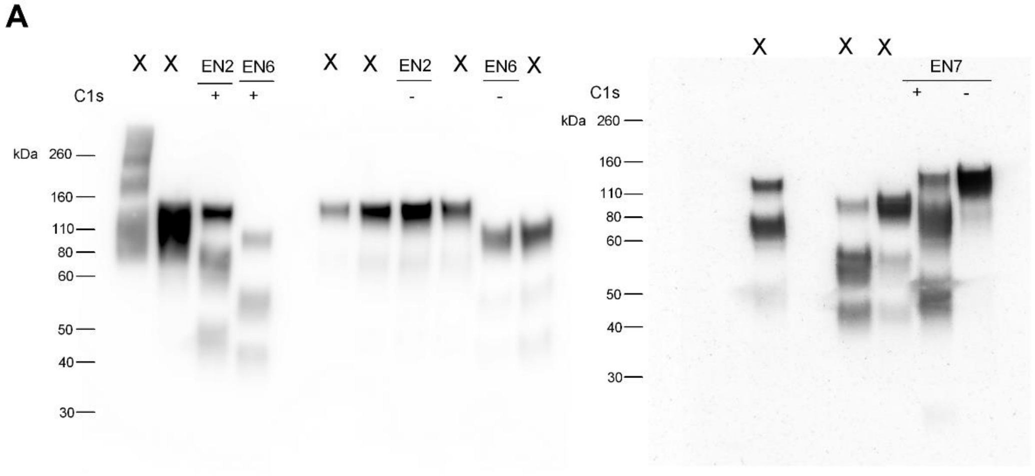

Figure. 3C. Western blot probed with anti-goat polyclonal conjugated to HRP.

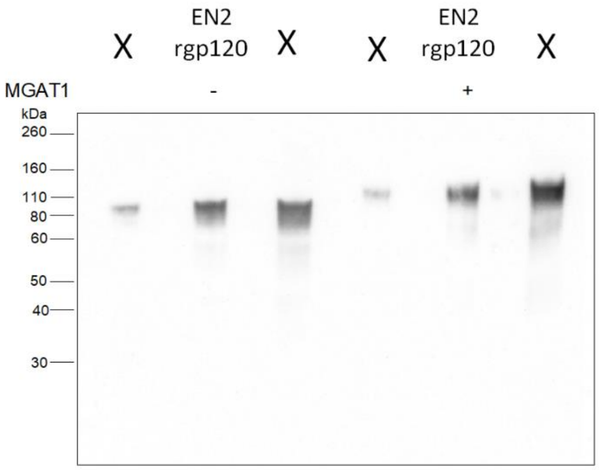

Figure 4C. Western blot probed with anti-goat polyclonal conjugated to HRP.

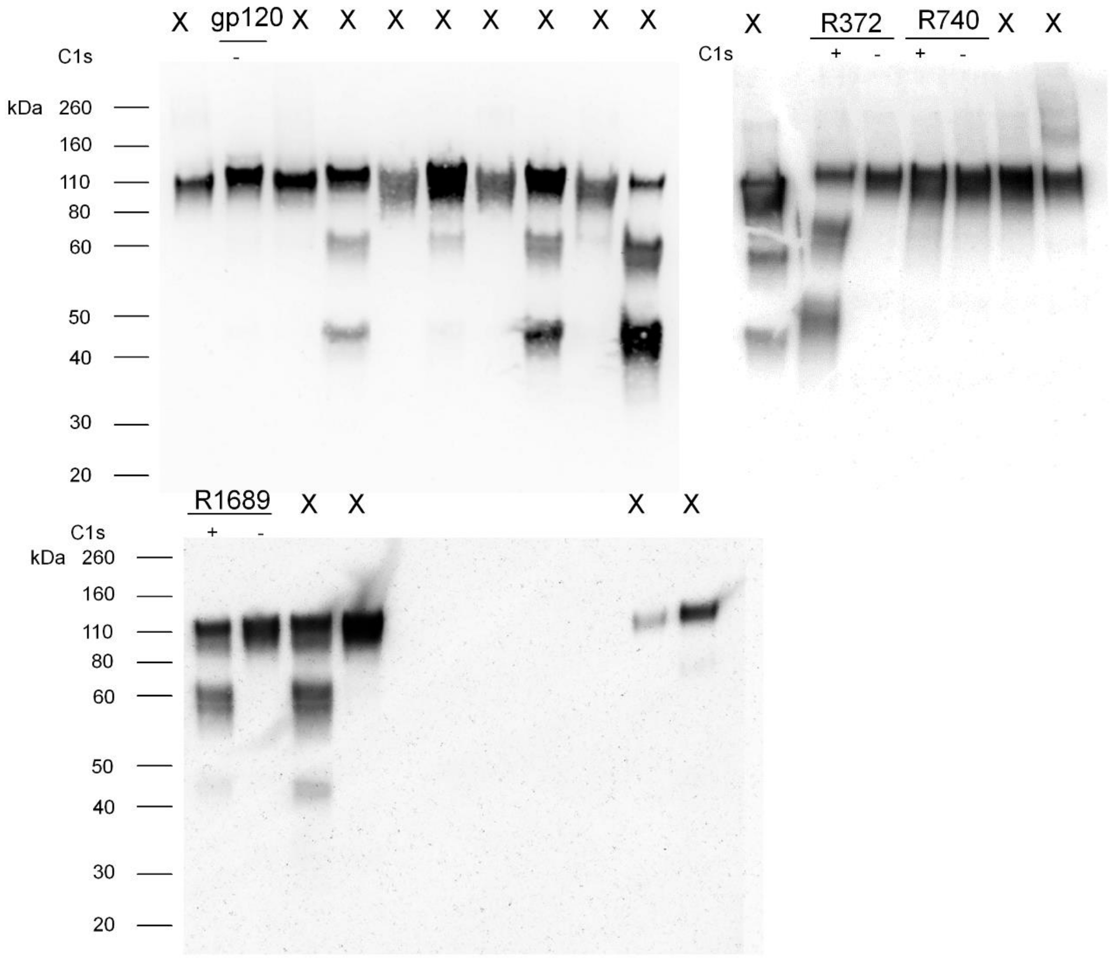

Figure 6B. Western blot probed with anti-goat polyclonal conjugated to HRP.

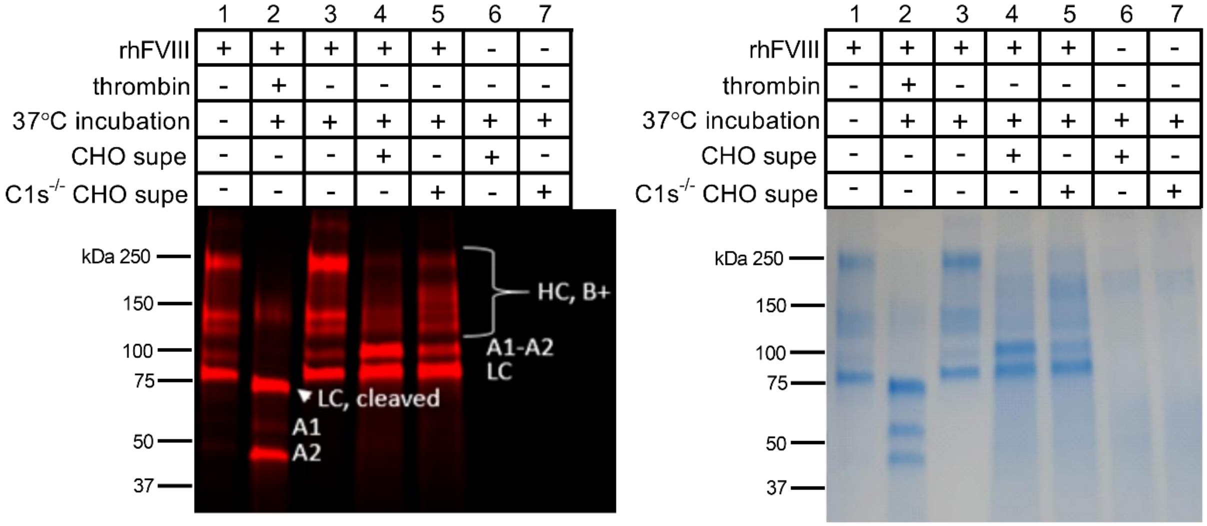

Figure 6C. Dylight 650 labeled Factor VIII and SDS-PAGE stained with Coomassie blue

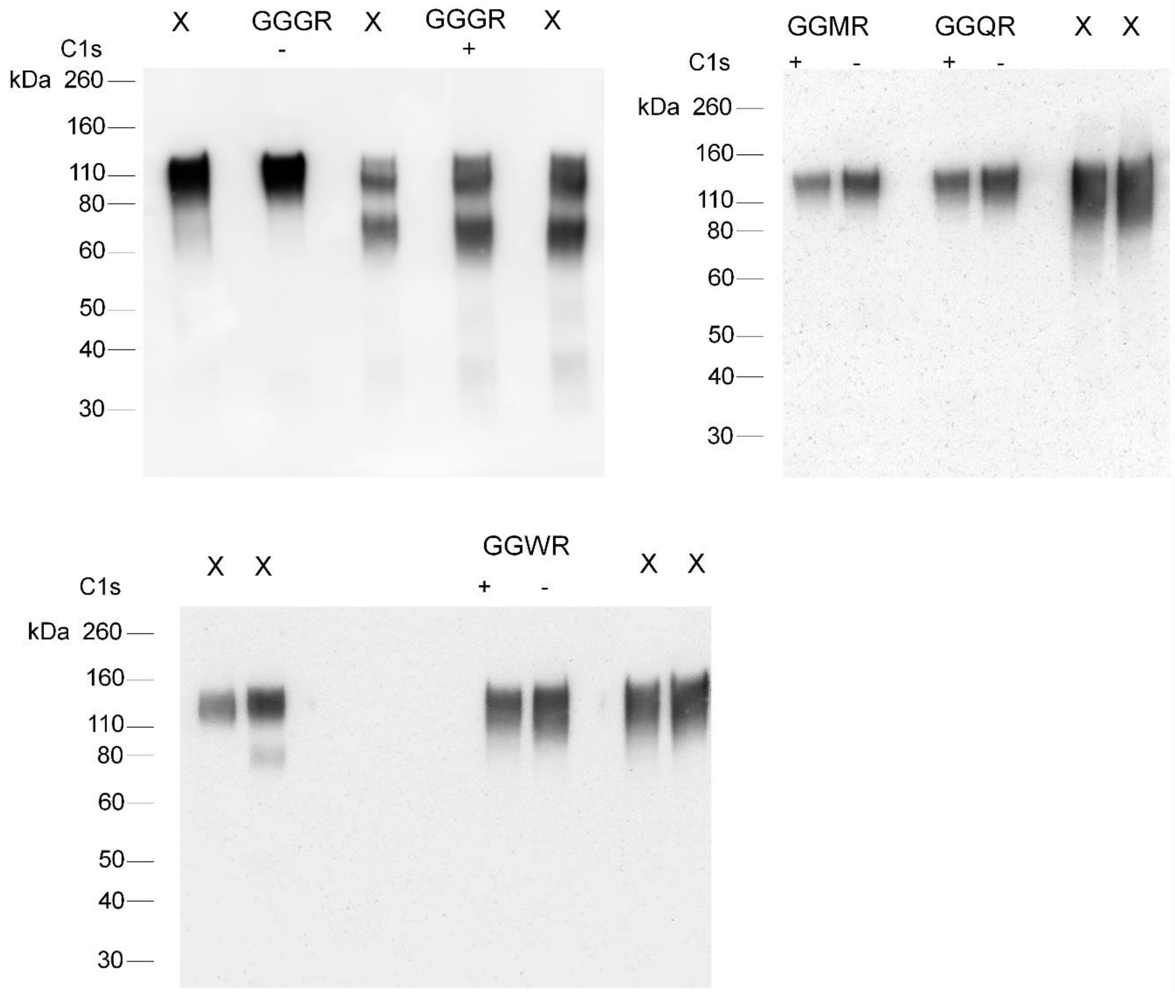

Figure 7B. Western blot probed with anti-goat polyclonal conjugated to HRP.
